# Awake Nasal Fiberoptic Intubation in Lateral Position for Severely Obese Patients with Anticipated Difficult Airway: A Randomized Controlled Trial

**DOI:** 10.3390/healthcare11212818

**Published:** 2023-10-24

**Authors:** Omar Ababneh, Isam Bsisu, Ahmad I. El-Share’, Mustafa Alrabayah, Ibraheem Qudaisat, Subhi Alghanem, Lubna Khreesha, Amani Mohamed Ali, Mohammad Rashdan

**Affiliations:** 1 Department of Anesthesia and Intensive Care, School of Medicine, The University of Jordan, Amman 11942, Jordan; m.rabayah@ju.edu.jo (M.A.); qudaisat@ju.edu.jo (I.Q.); alghanem@ju.edu.jo (S.A.); 2 UCSF Center for Health Equity in Surgery and Anesthesia, San Francisco, CA 94158, USA; 3 Department of Anesthesia and Pain Management, King Hussein Cancer Center, Amman 11941, Jordan; ahmad92shara@gmail.com; 4 Department of Special Surgeries, School of Medicine, The University of Jordan, Amman 11942, Jordan; l.khreesha@ju.edu.jo; 5 Department of Undergraduate Studies, School of Medicine, The University of Jordan, Amman 11942, Jordan; amani.ali2027@gmail.com; 6 Department of General Surgery, School of Medicine, The University of Jordan, Amman 11942, Jordan; m_rashdan@ju.edu.jo

**Keywords:** intubation, obesity, instrumentation, anesthesiology

## Abstract

Background: Obesity is a well-recognized risk factor for difficult intubation. To safely manage and overcome airway challenges in severely obese patients with a suspected difficult airway, awake fiberoptic intubation is recommended. We aimed to investigate the utility of awake nasal fiberoptic intubation in severely obese patients with suspected difficult airway while positioning them in the lateral decubitus position. Methods: This randomized controlled trial compared lateral and supine positions for awake nasal fiberoptic intubation in severely obese patients with an anticipated difficult airway by assessing the success rate, time needed to secure the airway, peri-procedural adverse events, and postoperative satisfaction of patients. Results: Sixty patients with a median age of 37 [inter-quartile range (IQR): 29–44] years were included, of which 47 (78.3%) were females. The median body mass index (BMI) was 45.5 [IQR: 42.5–50.8] kg/m^2^. The success rate of fiberoptic intubation was 100% in both groups. The time needed to successfully secure the airway was 188 [148.8–228.8] seconds (s) in the lateral position, compared to 214.5 [181.8–280.5] s in supine position (*p* = 0.019). Intraprocedural cough was more common in the supine position group (*n* = 8; 26.7%), compared to the lateral position group (*n* = 3; 10%; *p* = 0.095). Postoperative sore throat was more common in the lateral position group (*n* = 12; 40%) compared to the supine position (*n* = 5; 16.7%; *p* = 0.045). Conclusions: In conclusion, Intubation in the lateral position is a promising technique that is equivalent to the routine supine position during fiberoptic intubation. In fact, intubation in the lateral position took less time to successfully secure the airway.

## 1. Introduction

Obesity is a recurring chronic disease that is becoming increasingly prevalent worldwide. According to the World Health Organization (WHO), the majority of people now lives in countries where more deaths are caused by obesity than underweight [1]. Obesity is classified based on body mass index (BMI), with a BMI greater than 40 kg/m^2^ indicating severe obesity [2]. As obesity continues to spread, bariatric surgery is gaining popularity as a powerful treatment option [3,4].

Obesity is a well-known risk factor for difficult bag-mask ventilation and intubation [5,6], with an incidence of 11% for difficult intubation [7]. This can be attributed to physical characteristics such as a short neck, increased neck circumference, large tongue, excessive palatal and pharyngeal soft tissue, restricted mouth opening, large breasts, and limited cervical extension [8]. Although a high BMI does not always indicate a difficult airway [9], it is associated with rapid hypoxemia during airway manipulation due to reduced expiratory reserve volume (ERV) and functional residual capacity (FRC), as well as an increased risk for atelectasis even with the use of the ramping position and adequate pre-oxygenation [8,10,11].

To date, no single airway examination method is considered to be completely reliable in predicting difficult airways and intubation. Therefore, several tools and scoring systems have been developed by combining multiple tests to improve accuracy [12]. Among the individual tests used, neck circumference greater than 43 cm has been identified as an independent predictor of difficult intubation in obese patients, increasing the risk by 37% [13,14].

The El-Ganzouri risk index test was first published in 1996 and is still in use today; it uses a total score of 4 points as a cutoff in predicting difficult airway [15]. Subsequent papers set the score of 3–4 points as a significant cutoff in predicting a difficult airway that warrants the use of videolaryngoscopy, while scores higher than 7 mandate the use of fiberoptic intubation [16,17,18,19,20,21].

The lateral position decreases airway collapsibility in patients with obstructive sleep apnea (OSA) [22], making it easier to manage the airway in morbidly obese patients by maintaining airway patency [23,24]. In addition, the lateral decubitus position decreases the backpressure of abdominal organs on the chest, increasing the FRC without disturbing the ventilation perfusion (V/Q) matching in both lungs [25].

The lateral decubitus position has been previously recommended in airway management to maintain airway patency and to decrease risk of aspiration [26]. It has also been used to relieve airway obstruction when it occurs in the supine position [27,28]. Although anesthetists rarely do endotracheal intubations in the lateral position, interest in this technique has been previously addressed in the literature [26,29,30], as there is a daily risk of accidental extubation or urgent need to secure the airway in patients undergoing surgery in the lateral position, making it an important skill for an anesthesiologist to train for [31,32,33].

Previous studies have shown that intubation in the lateral position using the direct laryngoscopy technique led to a worse Cormack–Lehane laryngoscopic view compared to the supine position and an increase in time needed to secure the airway [30,34].

The use of fiberoptic intubation technology has significantly reduced failure rates of intubation [32,35]. Awake fiberoptic intubation is the safest approach in patients with anticipated difficult intubation, and the nasal route is the least discomforting approach in awake patients [36,37,38].

This study aims to investigate the utility of awake nasal fiberoptic intubation in severely obese patients with suspected difficult airway while positioning them in the lateral decubitus position. We compared the success rate of fiberoptic intubation and the duration needed to secure the airway between lateral and supine positions for awake nasal fiberoptic intubation in patients undergoing elective bariatric surgeries.

## 2. Materials and Methods

### 2.1. Study Design

**Figure 1:**
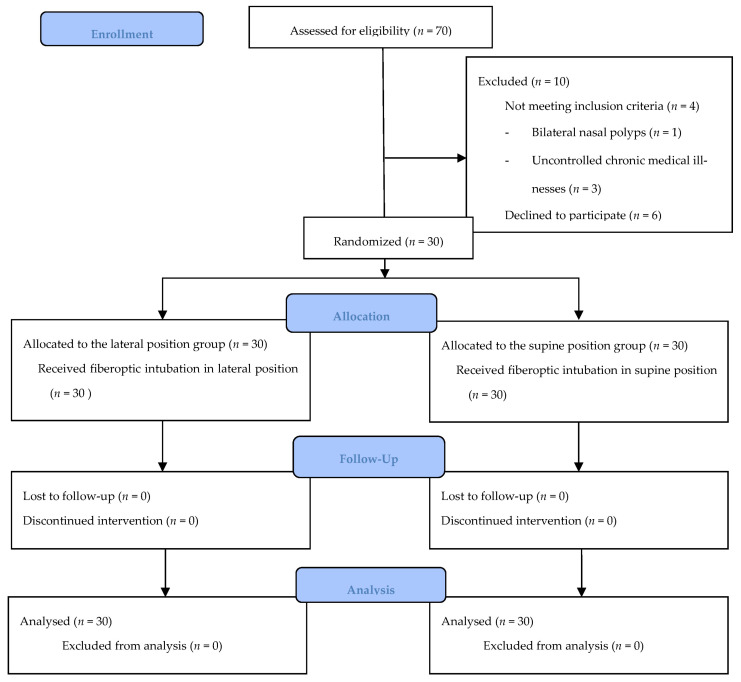
CONSORT flow diagram.

This prospective randomized controlled clinical trial was conducted at a tertiary teaching hospital, from March 2021 to January 2022. The study protocol was approved by the institutional review board (IRB) committee of Jordan University Hospital (442/2021/67) and was registered on ClinicalTrials.gov (Identifier: NCT04779528) on 3 March 2021. Written informed consent was obtained from patients prior to enrollment in the study and after explaining the procedure of awake fiberoptic intubation to them. The data were anonymized, and therefore no patient’s personal or identifying information was collected. The study followed the Declaration of Helsinki and Good Clinical Practice guidelines and was conveyed in accordance with the Consolidated Standards of Reporting Trials (CONSORT) statement (http://www.consort-statement.org/, accessed on 1 July 2023), as illustrated in Figure 1 (see Supplementary Materials).

### 2.2. Study Sample

We included patients who were severely obese (BMI > 40 kg/m^2^), had an American Society of Anesthesiologists Physical Status (ASA) Score of III [39], who did not have any other medical illnesses other than severe morbid obesity, and did not have a previously known allergy to the medications used in the study protocol. The included patients were undergoing elective bariatric surgery, namely laparoscopic sleeve gastrectomy and laparoscopic gastric bypass procedures. After assessment for difficult airways, we included patients with an El-Ganzouri Risk Index > 4 or neck circumference greater than 43 cm.

Exclusion criteria included non-consenting patients, an ASA score > III, coagulopathy, raised intracranial pressure, and patients with anatomical variations or pathological abnormalities leading to inability to perform nasal fiberoptic intubation. In the first random sample, we had one patient with large bilateral nasal polyps, as diagnosed by an ear, nose, and throat (ENT) surgeon. Thus, this case was replaced by another random subject.

We included a total of 60 patients after excluding non-eligible patients. The required sample size was calculated using Sample Size Calculator (http://riskcalc.org:3838/samplesize/#, accessed on 1 March 2021) [40]. We assumed a type I error rate (α) of 0.05 and power (1 − β) of 0.8 and an allowable mean difference of 45 s between lateral and supine positions to secure the airway in severely obese patients, with an expected population standard deviation of 60 s (1:1 enrollment ratio, drop rate 0%) [41]. The total sample size required was 60 patients, with 30 patients being enrolled in each group. None of the patients enrolled met our exclusion criteria or withdrew from the study.

Using GraphPad Prism 7.0 software (GraphPad Software Inc., San Diego, CA, USA), a computer-generated table was used to assign eligible patients randomly to two groups: supine position group (S) and lateral position group (L). To control bias, we provided detailed information about potentially outcome-affecting variables, including the demographics, airway exam, and preoperative assessment of the groups being compared, and there were no statistically significant differences between the two groups in the aforementioned variables.

### 2.3. Procedure

All patients received a standard preoperative assessment. The age, gender, weight, height, BMI, past medical illnesses, neck circumference, airway assessment, and El-Ganzouri Risk Index were recorded. Patients presented fasting for eight hours or more to the preoperative holding area, where no pre-medications were given. Baseline hemodynamic monitors were measured and recorded in the operating room according to standard ASA monitoring, and the bispectral index (BIS) was connected [42]. Peripheral intravenous access was established. The patient then was comfortably positioned according to the randomly allocated position and supported with pillows and arm rests. Patients were instructed to raise their arm if they were uncomfortable during the procedure.

The standardized method of moderate sedation and analgesia (sedoanalgesia) was applied on both groups during the awake fiberoptic intubation, with a targeted BIS score of 80 [43]. The sedation began by giving 2 mg of midazolam intravenously (IV), after which remifentanil intravenous infusion was started at a rate of 0.1–0.2 mcg/kg/min IV. The patients were reassessed after 2 min for the level of sedation, and we needed to adjust the medication dosages prior to proceeding with the fiberoptic intubation. Topical nasal lidocaine 2% gel was applied after the administration of intranasal decongestant and vasoconstrictor (which contains naphazoline hydrochloride and chlorpheniramine maleate). Two syringes were prepared for topicalization and application of local anesthesia through the fiberoptic scope during the procedure. Each contained 5 mL lidocaine 1% plus 5 mL air in a 10 mL syringe for application at the level of the vocal cords and then, after a 2 min waiting time, into the trachea.

A fiberoptic intubating scope sized 3.7 mm was loaded with a re-enforced endotracheal tube (size 6.5 mm for males, and 6.0 mm for females), and oxygen was supplied all through the procedure via conventional oxygen therapy methods. Patients were asked to point to which of their nostrils is more patent by assessing airflow through their nostrils following the administration of intranasal vasoconstrictor. The fiberoptic intubation procedure was carried out under standard ASA monitoring by a consultant anesthesiologist experienced in fiberoptic intubation and airway management. The scope was then passed through nasal passages via the more patent nostril. At the level of vocal cords, the preprepared 5 mL of lidocaine 1% was injected through the scope’s working channel port to anesthetize vocal cords. After waiting for two minutes, the scope was passed through the vocal cords toward the trachea, and then another 5 mL of lidocaine were injected through the scope’s working channel port to anesthetize the tracheal mucosa. After that, the endotracheal tube was slid forward until it was positioned 2 cm above the carina. In the lateral position group, the patients were asked to reposition themselves back into the supine position. Then, after securing the airway and ensuring correct connections of anesthesia machine circuit and the detection of CO_2_ via capnograph, general anesthesia was induced by a bolus of 2 mg/kg IV propofol, increasing basal remifentanil infusion to 0.5–1 mcg/kg/min IV and giving cisatracurium (0.15 mg/kg of ideal body weight) as a muscle relaxant.

### 2.4. Objectives

#### 2.4.1. Primary Objectives

The main objective was to compare the lateral and supine positions in terms of the success rate of awake nasal fiberoptic intubation, number of intubation trials, and duration needed to secure the airway. We counted each time the fiberoptic scope was entered through the nasal passages as an intubation trial. To measure the duration of the intubation procedure, we started a stopwatch from the beginning of the intubation procedure. We recorded T1 at the time of vocal cords; visualization and T2 when the airway was successfully secured.

#### 2.4.2. Secondary Objectives

1. Intraprocedural oxygenation and hemodynamic stability, airway complication, and sedation scores. To obtain these data, we documented the heart rate, electrocardiogram (ECG) rhythm, blood pressure, pulse oximetry, capnography, temperature, and BIS score every minute during the intubation procedure.
2. Postoperative airway-related complications (sore throat, cough, upper respiratory tract (URTI)-like symptoms, and epistaxis) 6 h after the end of surgery.
3. Patients’ satisfaction, which was measured using a 3-point Likert scale (pleasant, neutral, and unpleasant).

### 2.5. Statistical Analysis

We used the Statistical Package for the Social Sciences (SPSS) version 25.0 (Chicago, IL, USA) for data entry and the performance of statistical analysis. Descriptive statistics were performed, presenting the data as the number (percent) for categorical variables and the median and interquartile range (IQR) for continuous variables. A Kolmogorov–Smirnov test was used to test for normality of distribution. We then utilized the Mann–Whitney U test for the comparison between the two groups in terms of age, weight, height, BMI, ASA score, neck circumference, El-Ganzouri Risk Index for difficult airway, T1, T2, anesthesia time, surgical time, operating room time, and vital signs during the procedure. We then applied the Chi-squared test to compare categorical variables between the aforementioned two groups. A significance threshold of two-sided *p*-value < 0.05 was used for all aforementioned statistical tests.

## 3. Results

### 3.1. Demographics

Our sample included 60 patients in this study, with a median age of 37 [IQR: 29–44] years, of which 47 (78.3%) were females, while 13 (21.7%) were males. The median BMI of the studied population was 45.5 [IQR: 42.5–50.8] kg/m^2^. No significant differences were found between the two groups of the study in terms of the demographic data (Table 1). The type of surgery analysis showed that 40 (66.7%) patients underwent laparoscopic sleeve gastrectomy, and 20 (33.3%) underwent laparoscopic gastric bypass surgery.

The findings of the airway exam are summarized in Table 2. We found no significant differences between the two groups of the study. The median score for the El-Ganzouri Risk Index for difficult airway for the studied population was 4 [IQR: 4–5], with two patients scoring ≥7.

### 3.2. Analysis of Primary Objectives Variables

All patients in both groups were successfully intubated (100% success rate). Although there was no significant statistical correlation in T1 between the two groups (*p* = 0.679), T2 showed that the patients in the lateral position required 188 [IQR: 148.8–228.8] seconds (s) for endotracheal tube placement, while patients in the supine position needed 214.5 [IQR: 181.8–280.5] s (*p* = 0.019). The overall median number of trials was 1 [IQR: 1–1], with no significant difference between the two groups (*p* = 0.169). Only three (10%) patients in the lateral group required a second trial for intubation, compared to seven (23.3%) from the supine group. No significant differences were found between the two groups in terms of anesthesia time (*p* = 0.919), surgical time (0.744), or operating room time (*p* = 0.938; Table 3).

### 3.3. Analysis of Secondary Objectives Variables

We further investigated the vital signs, comparing them between the two groups from the start of the fiberoptic procedure until securing the endotracheal tube in place (Figure 1).

**Figure 2:**
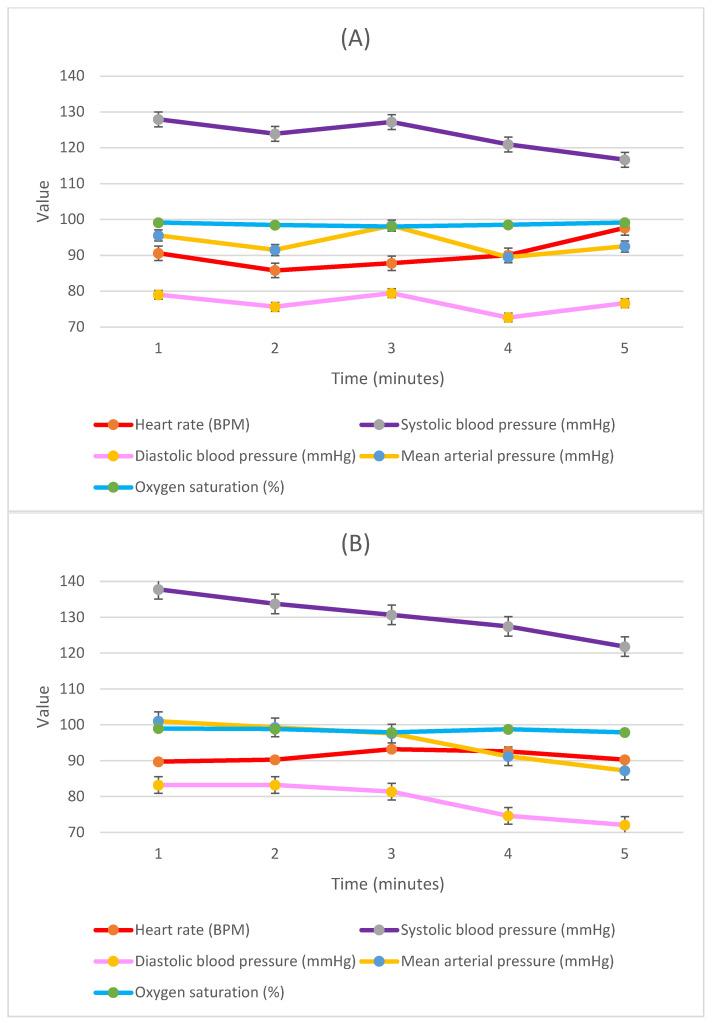
Hemodynamic parameters during first four minutes of awake fiberoptic intubation procedure in (**A**) lateral position and (**B**) supine position. The X-axis represents the time in minutes, while the Y-axis represents the values of measured vital signs; the heart rate is represented as beats/minute (BPM), blood pressure is presented in millimeter of mercury (mmHg), and oxygen saturation is represented as a percentage (%).

We found a significant difference in systolic blood pressure and mean blood pressure baseline upon positioning just prior to the start of the procedure, with a median of 127.0 [IQR: 121.3–137.0] mmHg for the baseline systolic blood pressure and median 95.5 [IQR: 87.8–104.5] mmHg for the baseline mean blood pressure of the lateral group, compared to 137.5 [IQR: 131.0–145.0] mmHg (*p* = 0.005) and 99.5 [IQR: 94.8–110.0] mmHg (*p* = 0.048), respectively, of the supine group. The systolic blood pressure was 127.0 [IQR: 111.8–134.5] mmHg for the lateral group and 131.5 [IQR: 123.0–147.0] mmHg for the supine patients after one minute (*p* = 0.037). The mean blood pressure at one minute also followed the same trend, with a median of 92.0 [IQR: 84.8–100.3] mmHg for the lateral group, compared to 97.5 [IQR: 91.0–110.3] mmHg for the supine group (*p* = 0.044). The rest of the vital parameters are presented in Figure 2, none of which showed significant statistical correlations.

When investigating the perioperative complications related to fiberoptic intubation, we found that intraprocedural cough was the most common adverse event during the procedure, and it was more common among those with the supine position (*n* = 8; 26.7%), compared to three (10%) patients among those with the lateral position (*p* = 0.095). We also found that minor periprocedural nasal bleeding developed among six (10%) of the patients in the total sample, with three (10%) being from each group. None of these incidents developed recurrent or more severe epistaxis over time and fiberoptic intubation was successfully completed for all of them. The postoperative follow-up showed that sore throat was the most common postoperative complication, and it was more common among those in the lateral group (*n* = 12; 40%), compared to those in the supine position group (*n* = 5; 16.7%), and this correlation was statistically significant (*p* = 0.045; Table 4).

We measured patients’ satisfaction and found that 61.7% of the total sample responded with pleasant, 28.3% responded with neutral, and 10% had an unpleasant experience. Both groups had comparable responses, and the statistical analysis showed no significant differences between the two groups (*p* = 0.95).

## 4. Discussion

Our main focus in this paper was the procedure of fiberoptic intubation in the lateral position compared to the supine position. We found that the lateral position required significantly less time to successfully secure the airway, with a mean of 188 s for endotracheal tube placement compared to a mean of 214.5 s in the supine position, making the difference 26.5 s between the two means. Reducing intubation time is considered an important aspect in advanced airway management, especially in patients with suspected difficult airways and difficult intubation who are at high risk for hypoxemia [44]. The 2022 ASA practice guidelines for the management of the difficult airway developed a difficult airway management approach that included awake airway management in patients with suspected difficult intubation and difficult ventilation when they are at an increased risk of aspiration, as well for those at risk of rapid desaturation [37]. They emphasized the awareness of the passage of time and limiting the number of attempts during difficult airway management [37].

The reduction in time needed to secure the airway may be due to the fact that the lateral position may decrease airway collapsibility and relieve airway obstruction when it occurs in the supine position [22,27,28], facilitating the management of the airway of morbidly obese patients by maintaining airway patency during the fiberoptic intubation procedure [23,24].

The differences in baseline blood pressure values between the supine and lateral groups may be due to the placement of the oscillometric blood pressure cuff on the non-dependent arm of patients in the lateral position, leading to lower readings compared to patients in supine position. Noninvasive blood pressure varies with changes in the arm and body position. In the lateral decubitus position, the non-dependent arm reads lower, when compared to the dependent arm, giving higher blood pressure readings [45]. Despite this difference, absolute values and the rate of change over time were within the normal range.

The most noteworthy adverse event was nasal mucosal injury during intubation, leading to epistaxis during or after the procedure. We had six cases of postoperative epistaxis (three from each group), and an ENT team was consulted for all cases, and all were mild and self-limiting without the need for further intervention. Epistaxis due to nasal mucosal abrasion is one of the commonest complications of nasal airway management and nasotracheal intubation, and in most cases, it is a self-limiting complication [46,47]. Several protective measures have been recommended to control this adverse event, such as the application of local vasoconstrictive drugs, warming and softening of the endotracheal tube, and the use of the nasopharyngeal airway as a pathfinder. However, this adverse event cannot be avoided entirely [48].

Intraprocedural cough was significantly more common in supine patients (26.7%), compared to 10% in the lateral position. This may be due to the fact that the lateral position helps to clear secretions away from sensitive midline structures of the airway, such as epiglottis and carina.

The most common side effect was a postoperative sore throat, which was significantly more common in the lateral position group (40%) versus (16.7%) in the supine position group. This may be due to the movement of the cuff and tube within the trachea during patient repositioning after intubation, which has been linked to postoperative sore throat [49].

We aimed to maintain adequate anxiolysis during the procedure, and all patients were instructed to raise their hand if they become uncomfortable to notify the anesthesiologist. Concurrently, vital signs and BIS scores were continuously measured (as demonstrated in Figure 1). This suggests that sedation was sufficient in both groups to allow for successful fiberoptic intubation while maintaining vital signs within the normal range. The stability of blood pressure and heart rate also indicated adequate anxiolysis and patient comfort.

Patients’ postoperative satisfaction showed that both groups had comparable results, with 61.7% of patients rating the procedure as a pleasant experience. This indicates that minimal anxiolysis and moderate sedation with analgesia are adequate for the awake fiberoptic intubation procedure without the need to deepen the level of anesthesia, which may lead to respiratory depression and hypoxemia in this high-risk group of patients.

Finally, we conducted this study on severely obese patients who do not have any other chronic medical illnesses. Many anesthesiologists may not be familiar with how to deal with an anesthetic crisis, such as cardiac arrest, in the lateral position [50]. Moreover, some physiological changes in the lateral position may not be suitable for all patients. This disadvantage might limit the utility of lateral position in patients who are at a high risk of experiencing a perianesthetic crisis. Therefore, further investigations are recommended, and the anesthesiologists and operating room staff must be trained on how to deal with such scenarios prior to adopting this technique, and they must be trained on how to revert back to the supine position in a time efficient way when needed.

This study had several limitations. We excluded patients with anatomical or pathological abnormalities that led to the inability to perform nasal fiberoptic intubation. Obesity can be associated with hypertrophy of nasopharyngeal soft tissue and polyps’ formation, with increased risk for further nasal mucosal injury and obstruction with nasal fiberoptic intubation trials [51]. Furthermore, the small sample size may have undermined the external validity of a study. Moreover, the anesthesiologists performing the procedure were experienced in fiberoptic intubation techniques, so the results may not apply to resident physicians and anesthesiologists who are not experienced in fiberoptic intubation.

## 5. Conclusions

With the advancement of anesthesia monitors, equipment, and medications, anesthesia is becoming safer, and non-routine techniques are being re-explored and studied. The lateral position has great utility in patients with anticipated difficult airways, and in our paper, we found it safe and equivalent to routine supine position during awake fiberoptic intubation, with a success rate of 100% on both groups. The lateral position also required less time to successfully secure the airway, and patient satisfaction scores were comparable to those of patients intubated in supine position. The applicability of intubation in lateral position also allows patients to choose the position that is most comfortable for them during awake fiberoptic intubation. This study was performed on severely obese patients undergoing elective surgeries, and the utility of the lateral position for fiberoptic intubation in emergency situations is yet to be studied, especially that in emergency cases, when airway management might be critical, and further research is needed to determine the usefulness of the lateral position in these situations.

## Figures and Tables

**Table 1:** Demographics of the studied population.

Characteristic	Lateral Position (*n* = 30)	Supine Position (*n* = 30)	Total (*n* = 60)	*p*-Value
Age (years)	39 [32–42.3]	35 [26–46]	37 [29–44]	0.657
Gender				
Male	7 (23.3%)	6 (20%)	13 (21.7%)	0.754
Female	23 (76.7%)	24 (80%)	47 (78.3%)	
Weight (kg)	122 [111–143]	120.5 [110–149]	121.5 [111–143]	0.929
Height(cm)	165 [157.8–170.8]	162.5 [153.75–170]	165 [156–170]	0.387
BMI	45.5 [42–52]	45.5 [42.9–50.5]	45.5 [42.5–50.8]	0.717
ASA score	3 [3–3]	3 [3–3]	3 [3–3]	0.643
Allergies				
NKDA	29 (96.7%)	29 (96.7%)	58 (96.7%)	1
Penicillin allergy	1 (3.3%)	1 (3.3%)	2 (3.3%)	

BMI, body mass index; ASA score, American Society of Anesthesiologists Physical Status Score; NKDA, no known drug allergies; kg, kilograms; cm: centimeters. Numbers are presented as number (percent) and median [inter-quartile range (IQR)].

**Table 2:** Airway examination findings in the studied population.

Characteristic		Lateral Position (*n* = 30)	Supine Position (*n* = 30)	Total (*n* = 60)	*p*-Value
Mouth opening (cm)	≥4 fingers	26 (86.7%)	26 (86.7%)	52 (86.7%)	1
	<4 fingers	4 (13.3%)	4 (13.3%)	8 (13.3%)	
Thyromental distance (cm)	>6.5 cm	21 (70%)	23 (76.7%)	44 (73.3%)	0.826
	6.0–6.5 cm	6 (20%)	5 (16.7%)	11 (18.3%)	
	<6.0 cm	3 (10%)	2 (6.7%)	5 (8.3%)	
Mallampati score	I	0 (0%)	1 (3.3%)	1 (1.7%)	0.145
	II	10 (33.3%)	4 (13.3%)	14 (23.3%)	
	III	19 (63.3%)	25 (83.3%)	44 (73.3%)	
	IV	1 (3.3%)	0 (0%)	1 (1.7%)	
Neck movement	>90	27 (90%)	27 (90%)	54 (90%)	1
	80–90	3 (10%)	3 (10%)	6 (10%)	
	<80	0 (0%)	0 (0%)	0 (0%)	
Ability to prognath	Yes	25 (83.3%)	24 (80%)	49 (81.7%)	0.739
	No	5 (16.7%)	6 (20%)	11 (18.3%)	
Body weight group (kg)	<90	1 (3.3%)	0 (0%)	1 (1.7%)	0.307
	90–110	2 (6.7%)	5 (16.7%)	7 (11.7%)	
	>110	27 (90%)	25 (83.3%)	52 (86.7%)	
History of difficult intubation	None	30 (100%)	29 (96.7%)	59 (98.3%)	0.313
	Questionable	0 (0%)	1 (3.3%)	1 (1.7%)	
	Definite	0 (0%)	0 (0%)	0 (0%)	
Neck circumference (cm)		42 [39–45.3]	42.5 [39.4–46]	42 [39.1–45.8]	0.941
El-Ganzouri = ∑		4 [4–5]	4 [4–5]	4 [4–5]	0.440

∑, total; kg, kilograms; cm, centimeters. Numbers are presented as number (percent) and median [inter-quartile range (IQR)].

**Table 3:** Intraoperative data and airway management.

Characteristic	Lateral Position (*n* = 30)	Supine Position (*n* = 30)	Total (*n* = 60)	*p*-Value
Procedure				
Laparoscopic sleeve gastrectomy	19 (63.3%)	21 (70%)	40 (66.7%)	0.584
Laparoscopic gastric bypass	11 (36.7%)	9 (30%)	20 (33.3%)	
Nostril				
Left	15 (50%)	13 (43.3%)	28 (46.7%)	0.605
Right	15 (50%)	17 (56.7%)	32 (53.3%)	
Length at which ETT was fixed (cm)	25 [24–26]	25 [25–26]	25 [24–26]	0.563
T1 (seconds)	28 [21.3–42]	30 [21–46]	29 [21–44.5]	0.679
T2 (seconds)	188 [148.8–228.8]	214.5 [181.8–280.5]	201.5 [166.3–243.8]	0.019
Number of trials	1 [1–1]	1 [1–1.3]	1 [1–1]	0.169
Anesthesia time (minutes)	106 [80.3–125]	106 [81.8–127.8]	106 [81–126.3]	0.919
Surgery time (minutes)	84.5 [58.5–109.8]	72.5 [56.5–100.8]	80.5 [57.8–103.5]	0.744
Operating room time (minutes)	117 [88.5–140]	116 [93–139.3]	117 [92.3–140]	0.938

T1, time of vocal cords visualization; T2, time when the airway was successfully secured; ETT, endotracheal tube; cm: centimeters. Numbers are presented as number (percent) and median [inter-quartile range (IQR)].

**Table 4:** Postoperative side effects related to the fiberoptic intubation.

Characteristic	Lateral Position (*n* = 30)	Supine Position (*n* = 30)	Total (*n* = 60)	*p*-Value
Intraprocedural				
Intraprocedural cough	3 (10%)	8 (26.7%)	11 (18.3%)	0.095
Minor periprocedural nasal bleeding	3 (10%)	3 (10%)	6 (10%)	1
Postprocedural				
Sore throat	12 (40%)	5 (16.7%)	17 (28.3%)	0.045
URTI-like symptoms	2 (6.7%)	0 (0%)	2 (3.3%)	0.492
Nausea	1 (3.3%)	1 (3.3%)	2 (3.3%)	1
Epistaxis within 6 h postoperatively	1 (3.3%)	4 (13.3%)	5 (8.3%)	0.353
Satisfaction				
Pleasant	19 (63.3%)	18 (60%)	37 (61.7%)	0.958
Neutral	8 (26.7%)	9 (30%)	17 (28.3%)	
Unpleasant	3 (10%)	3 (10%)	6 (10%)	

URTI: upper respiratory tract infection. Numbers are presented as number (percent).

## Data Availability

The data presented in this study are available upon request from the corresponding author.

## Supplementary Materials

The following supporting information can be downloaded at https://www.mdpi.com/article/10.3390/healthcare11212818/s1, File S1: CONSORT 2010 checklist of information included when reporting this randomized trial.
